# Design of Fuzzy Logic Motion Detection Algorithm for the Bracelet Type Sensor Consisting of Conductive Layer-Polymer Composite Film

**DOI:** 10.3390/polym14122309

**Published:** 2022-06-07

**Authors:** Kiwon Park

**Affiliations:** Department of Mechanical & Automotive Engineering, Youngsan University, Junam-ro 288, Yangsan-si 48015, Korea; kwp@ysu.ac.kr

**Keywords:** carbon-based conductive layer-polymer composite film, fuzzy logic motion detection algorithm, sensor mechanism, sensor fabrication, bracelet type sensor

## Abstract

To improve the motion detection performance of a bracelet-type sensor that uses only two tiny sensor modules developed using carbon-based conductive polymer composite films, a fuzzy-logic algorithm was developed in this study. A polyethylene terephthalate polymer film with a conductive layer composed of carbon paste was used as the integral material utilized for the composite film; a small sensor module composed of mechanical parts mounted on the film was developed to effectively detect the surface resistance variations of the film. A participant wore a bracelet sensor, which consisted of two sensor modules, on their forearm, and the resistance variations of the contact area between the forearm and the sensor modules corresponding to the flexion changes of the surface of the body due to muscle contraction and relaxation were detected. The surface resistance variations of the film were converted to voltage signals, which were used as inputs to the fuzzy logic algorithm to detect four consecutive motions of the forearm. The results demonstrated that the fuzzy-logic algorithm attained an accuracy of 94%. The fuzzy algorithm successfully detected four motions and the resting state of the forearms; moreover, it showed improved performance compared to previous research.

## 1. Introduction

Recently, wearable devices have been applied in numerous fields because they enable the use of physiological and internally and externally produced information in real-time.

One of the important technologies in wearable devices involves the detection of human movement intention [1], which enables a variety of applications, such as prosthetic device control, sports, entertainment, and various creative applications, by connecting with the Internet of Things (IoT). Therefore, a variety of sensors have been utilized to develop effective wearable sensors. The conventional method is to use a patch type electrode attached on the skin to measure the body’s surface electromyography (sEMG) signals. The sEMG supplied the overall synergistic activity data of a set of muscular units for quantitative analysis to detect the intention of movement. However, the noises caused by the movements of other muscles and the effects inside and outside the body, such as muscle fatigue and changes in attachment position disturb the effective detection of body movement. Therefore, signal processing techniques are required to extract useful signals from the sEMG. Moreover, sEMG measurement requires multiple electrodes attached to the surface of the body because at least three electrodes must be attached; among which, two electrodes are for differential amplification and one for the ground. Owing to the requirement of multiple electrodes in sEMG measurement, bracelet-type sensors using sEMG have restrictions in terms of size reduction and are therefore fabricated in the shape of a wide armband [2,3,4].

To overcome these inconveniences of using sEMGs, researchers have been developing wearable sensor systems to detect the motion intention of the body without using sEMG signals. Most studies have used commercially available sensors such as strain gauges [5], tactile sensors [6,7,8,9,10], and acceleration sensors [11,12] to develop sensor systems that can detect human movement.

There have been studies on the development of sensor materials that can be applied to wearable sensors. A strain gauge element with a specific pattern was fabricated for movement detection and strain measurement [13,14,15]. To develop an elastic strain gauge sensor, researchers have used liquid-phase metals [16,17,18,19,20] and force sensors to detect the expansion pressure by muscle contraction to detect the movement intention [21,22]. Several types of sustainable composite materials of self-sufficient power sources exist, converting energy from the environment into electrical energy. An example of such materials is triboelectric composite materials, which are suitable for wearable sensor applications. Triboelectric materials show good response under high-frequency stimuli [23]. Therefore, they have been used for developing acceleration sensors to detect different motions, such as sitting, standing, and walking [24]; they have also been used as energy harvesting nanogenerator from body movements corresponding to high-frequency stimuli [25].

Studies on the development of band-type sensors using commercial or developed sensor elements have been reported. Commercial pressure and inertial sensors have been applied to detect hand gestures and the arm and leg motions, respectively [11,12]. An ionic liquid-based fluidic strain sensor was developed and worn on a leg and fingers to detect movements [26]. A flexible optical pressure sensor was developed and worn on index finger to detect flexion and extension of it [27].

**Figure 1:**
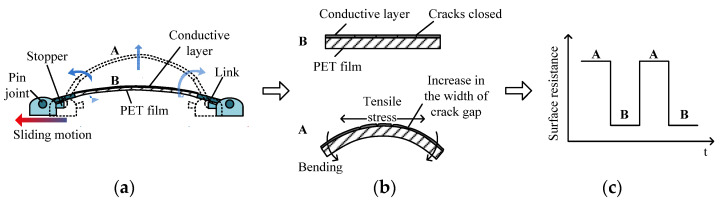
Description of the sensor mechanism: (**a**) Bending generation mechanism, (**b**) Relationship between the bending of film and the increase in the width of crack gaps, and (**c**) Illustration of resistance variation on the conductive layer.

In a previous study, a thin bracelet-type wearable sensor module, which uses a carbon-based conductive layer-polymer composite film to detect the movement intention of the human body, was developed; a comprehensive study was conducted to verify the sensor performance as a wearable sensor [28]. The integral material utilized for the composite film is 0.1 mm thick PET (polyethylene terephthalate) polymer film with a conductive layer made using a carbon paste and the heat press process, which is sufficiently flexible to detect the changes in the resistance corresponding to the flexion changes of the body surface due to muscle contraction and relaxation. The detailed fabrication procedure for the composite films can be found in ref. [28]. Figure 1 shows the mechanism of surface resistance variation of the polymer film. The width changes in the fine (<10 μm) crack gaps in the conductive layer play a key role in the surface resistance variations corresponding to the bending motion of the film, which was investigated in ref. [29]. As shown in Figure 1a, the sliding movement of the mechanical parts holding each tip of the film occurs owing to the contraction and extension of the inner muscles of the contact area, which causes the bending motion of the film (state A). When the film was bent, tensile stress was applied to the conductive layer of the film, and the width of the crack gaps increased, resulting in an increase in surface resistance (Figure 1b). When the film returned to state B, the cracks closed, decreasing the surface resistance (Figure 1c). A detailed explanation of the relationship between the variation in crack gaps and surface resistance variation with the images of generated cracks can be found in refs. [28,29].

**Figure 2:**
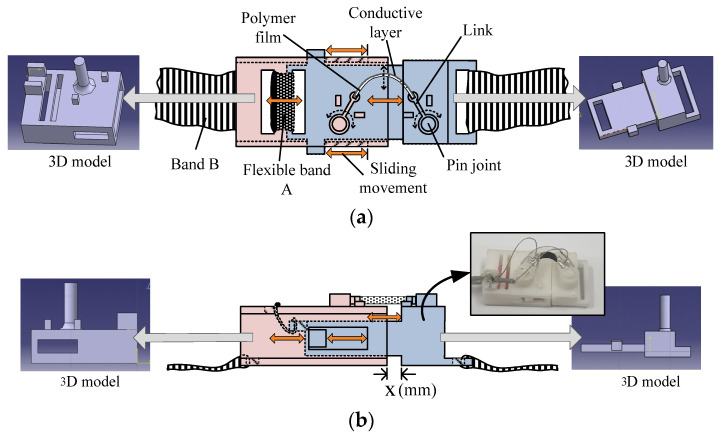

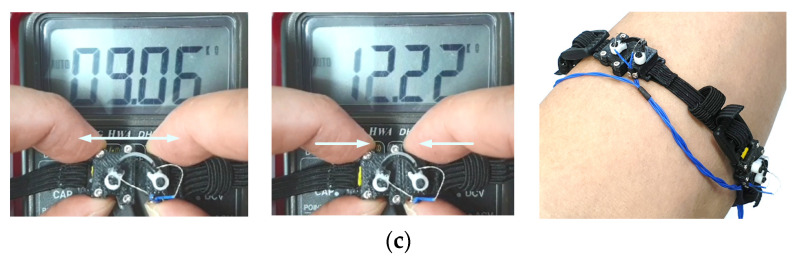
Movement mechanism and fabricated form of sensor module: (**a**) Top view, (**b**) Side view with fabricated image of module, (**c**) Voltage signal variation due to the change in the surface resistance of the composite film mounted on the sensor module and the image of final version sensor modules when worn with permission from [28]. 2022 MDPI Publishing. (K. Park, Sensors, vol. 22, 2022, MDPI Publishing).

The fabricated film was cut into pieces of dimensions 2 (w) × 18 (l) (mm). Because the thin film is easily damaged by the surrounding effects, a mechanical module that protects the film from environmental effects and guides its bending action without degradation of the conductive layer was developed, and the film was mounted on the module. Figure 2a,b shows the structure and fabricated image of the sensor module comprising the two connected parts. The distance between each part of the module increases or decreases during the sliding motion, where the moving distance (x mm in Figure 2b) is limited from 0 to 3 mm. When the distance between two parts is 0 mm, the dimensions of a mechanical module are 12.0 (w) × 30.5 (l) × 5.0 mm (h). Corresponding to the flexion changes of the contacting surface of the body, the mechanical module converts its stretching and shrinking motions to the bending motion of the mounted film. In addition, the mechanical module limits the application of an excessive compressive force on the conductive layer of the film because the compressive force degrades the conductive layer, resulting in a decrease in the reproducibility of the sensor. In previous research, compressive forces caused more severe degradation of the surface conductive layer compared to the tensile forces. A detailed explanation of the conductive layer degradation mechanism under compressive and tensile forces can be found in [29]. To fabricate the bracelet-type sensor, two sensor modules were connected to each other using a flexible bend with a width of 9 mm and worn on the forearm. Figure 2c shows the variation in resistance owing to the sliding movement of the mechanical parts in the module and the worn image of the bracelet-type sensor. The variations in the surface resistance measured by the bracelet sensor are converted into voltage signals through a transducer circuit, and they are measured to detect the motions of the forearm. A more detailed explanation of the fabrication method of the film and bracelet sensors is provided in ref. [28].

The developed sensor system had several advantages. First, a single, thin bracelet-type sensor can be worn around the forearm instead of the conventional patch sensors used in the sEMG method, which would cover most parts of the forearm. As a result, compared to the conventional sEMG technique, the developed sensor enables a reduction in the number of sensors contacting the skin surface by more than 70%. Therefore, the proposed sensor should be comfortable in terms of wearability and practical for various applications. Second, conductive paste and PET film, which are easier to handle and more cost effective than other approaches that use carbon materials such as CNTs [30,31,32,33,34] are the materials used in this study. Finally, because a highly sensitive signal is produced by the simple bending motion of the polymer film, the proposed method has a wide application area with various types of mechanical guides in wearable sensor technology to detect body movements.

Although the developed bracelet sensor shows promising performance, there are drawbacks that must be improved before practical use. In the previous study, a simple algorithm, comprising of ‘if and else’ statements, was used and a motion detection test for four motions of the forearm was conducted for a single motion at a time. However, wearable sensors should be able to detect a variety of motions occurring continuously in real applications. Therefore, further research to develop improved algorithms should be conducted to detect various consecutive motions over a long period of time.

To improve motion detection performance, the algorithm should be able to classify the complex and nonlinear signals generated from the complex structure of the human body. Fuzzy logic is extensively used in the motion-detection technique [35,36,37,38,39,40,41].

In this study, a fuzzy logic algorithm was developed to detect four motions of the forearm, wrist extension, ulnar deviation, finger flexion, and wrist flexion, and the performance verification of the algorithm focuses on the detection of the consecutive motions. Moreover, it is possible to decrease the number of sensor modules in the bracelet sensor when the designed fuzzy logic algorithm is applied, resulting in a simplified structure of the bracelet sensor. In a previous study, the performance of a bracelet sensor using two and three sensor modules was compared, and the bracelet sensor with three sensor modules showed better performance [28]. However, in this study, a bracelet sensor with two sensor modules was used with the fuzzy motion detection algorithm. In the experiment, the detection performance of 24 combinations of four consecutive motions was conducted, and it showed excellent performance in detection of a single motion as well as in the detection of consecutive motions.

## 2. Experimental Setup

**Figure 3:**
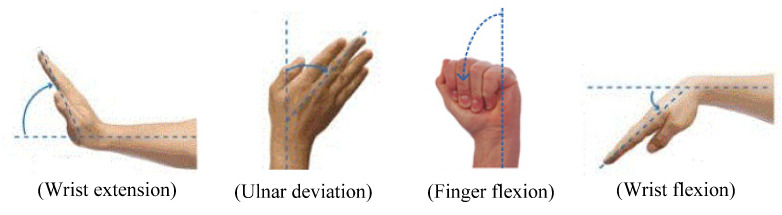
Forearm movements.

In this study, the same experimental procedure as that used in [28] was applied. However, the bracelet sensor used in this study consists of only two sensor modules. Figure 2 shows the structure and an image of the fabricated sensor. The fabricated sensor module had sufficient sensitivity to detect even smaller flexion variations on the surface of the human body. A graph showing the sensitivity of the resistance variations of the composite film corresponding to the stretched length of each module, which is represented by x (mm) in Figure 2b, can be found in [28]. In addition, the reproducibility and repeatability data during 120 bending cycles, where each cycle consisted of 11 bending motions below 90°, can be found in [29]. To detect the four motions of the forearm, wrist extension, ulnar deviation, finger flexion, and wrist flexion shown in Figure 3, the bracelet sensor was worn such that the two sensor modules contacted the surface of the forearm muscle groups: extensor carpi ulnaris and flexor carpi ulnaris.

As shown in Figure 2c, the sensor module stretches in the longitudinal direction and the conductive layer resistance decreases when the muscles contract, resulting in skin protrusion. Conversely, the sensor module contracts in the longitudinal direction, and the resistance of the conductive layer increases when the muscles expand, resulting in depression of the skin surface.

Because the band connecting the two mechanical parts in the sensor module (band A, Figure 2a) exhibits lower elasticity than the band connecting the sensor modules (band B, Figure 2a), the protrusions and depressions can be effectively detected by the sensor module.

**Figure 4:**
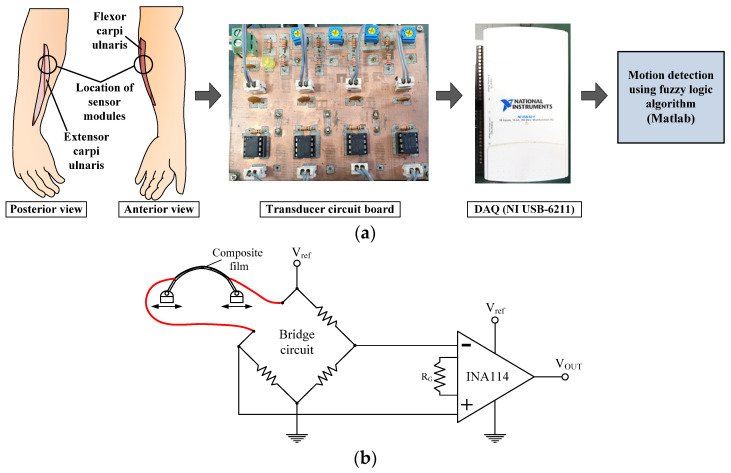
Experimental setup and Transducer circuit converting resistance variation to voltage signal: (**a**) Composition of data acquisition procedure, (**b**) Transducer circuit.

Figure 4a shows the data acquisition procedure for detecting the forearm motion using the fuzzy logic algorithm, and Figure 4b shows the transducer circuit.

The present study only uses two sensor modules comprising a bracelet sensor developed to measure the signals generated by the four motions. However, the conventional sEMG method to detect the movement of the body requires two electrodes to be attached on each muscle group to measure the charge of sEMG signals; therefore, nine electrodes, including one ground electrode, are required to be attached to detect the four motions of the forearm, on the locations of the muscle group, which govern each motion.

Figure 4b shows the transducer circuit, which consists of a bridge circuit and differential amplifier IC (INA114) for each module to convert the surface resistance variations to voltage signals. A 50-KΩ R_G_ is used to obtain amplification by a factor of 50. The signals transmitted by each sensor module on the upper arm were collected using an NI-DAQ with a sampling frequency of 1 kHz. In this study, 0.1-μF capacitors were used to remove high-frequency noises.

The signal was measured for 30 s. During the first 5 s of data collection for each movement, the forearm rested on the table without any movement. Subsequently, four consecutive motions were performed, where each motion was maintained for approximately 5 s, after which a resting state was maintained for the remaining 5 s. During all the combinations of the four motions, a total of 24 data sets of the measured signals were gathered. All movements were performed at a constant speed throughout the entire range of motion of the joint.

The measured datasets were loaded into MATLAB, and motion detection was conducted using the designed fuzzy logic algorithm. Although the developed sensor exhibited good performance, as proven by previous studies, it was difficult to acquire constant signals for the same motion owing to variations in factors such as muscle fatigue, strength, and speed of motion. Therefore, the fuzzy rules in the algorithm were adjusted for each motion set to obtain the optimal result.

## 3. Design of Fuzzy Logic Motion Detection Algorithm

**Figure 5:**
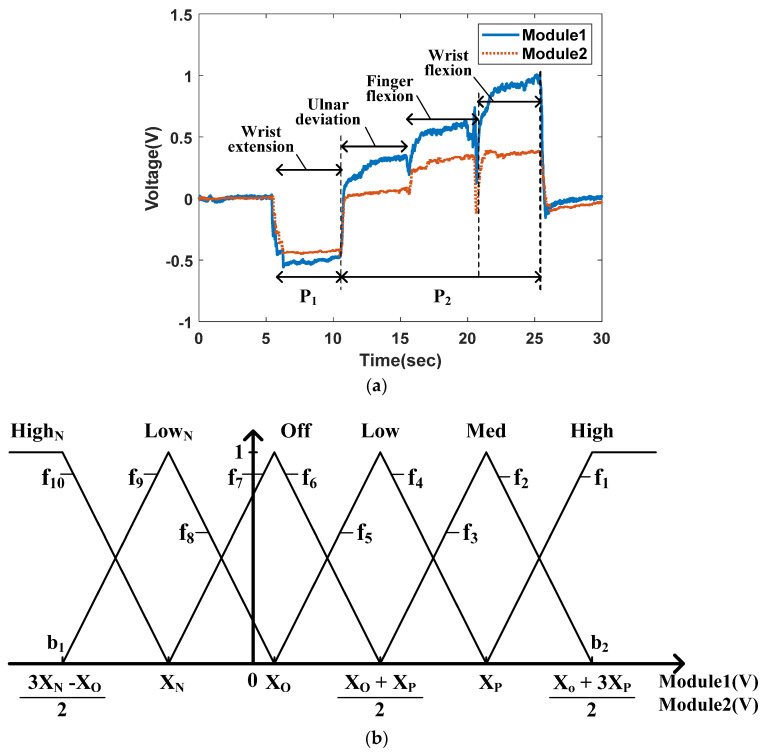
Relationship between the measured signal and the input membership function: (**a**) Measured signal data from module 1 and 2, (**b**) Input membership function.

A fuzzy logic system generally consists of three-step data processing: fuzzification, rule evaluation, and defuzzification. First, the fuzzification phase converts the measured data from the two sensor modules into fuzzy data with the names (High_N_, Low_N_, Off, Low, Med, High in Figure 5b) and weight from zero to one. This process is conducted using the input membership function. Second, the rule evaluation phase uses the ‘*if*–*then*’ conditional statement relating the fuzzy values from the input membership function to the level of outputs in Table 1. Third, the defuzzification process estimates the motion of the forearm using the weights of all active rules (with nonzero weights generated by the input triangular functions (f_1_–f_10_ in Figure 5) and the center values. All fuzzy logic processes were conducted using MATLAB (Ver. R2019a).

### 3.1. Fuzzification

Through the fuzzification process, the mean of every five measured data points from modules 1 and 2 located on two muscle groups, extensor carpi ulnaris and flexor carpi ulnaris, are sequentially reclassified into weighted fuzzy data using the input membership function. Figure 5a shows the measured data from modules 1 and 2, while four consecutive motions of the forearm, wrist extension, ulnar deviation, finger flexion, and wrist extension were performed, where each motion was maintained for approximately 5 s. Figure 5b shows the input membership function, where *X_N_* is the mean of all the negative data values appearing during the wrist extension motion, marked as P_1_ in Figure 5a. *X_P_* is the mean of the positive data that appears during the other three motions, marked as P_2_ in Figure 5a. The *X_O_* is the mean of the data measured during the resting state. During the fuzzification process, two input membership functions are used for each data point. The membership functions comprise trigonometric functions consisting of six names (High_N_, Low_N_, Off, Low, Med, and High) according to the input range, and each waveform is configured to have a 50% overlap. In each fuzzification step, the means of five datasets from modules 1 and 2 are classified as names corresponding to the range where the data belong, and each name has a weight value ranging from zero to one. Because two trigonometric functions overlap in the range between the boundaries (b_1_ and b_2_ in Figure 5b) on the *x*-axis, the two names have weight values, while the other four names have ‘0’ weight values. Depending on where the data belong, the data exceeding the boundaries, the name of the fuzzy data is either ‘High_N_’ or ‘High’, and the weight of name is fixed as ‘1’, and other five names have ‘0’ weight values.

Algorithm 1 shows the MATLAB code algorithm used to realize fuzzification using the input membership function in Figure 5b. Because the code generates six fuzzy names and their own weights from the signal measured by a single module, 12 fuzzy values with each name and weight are generated in every fuzzification step.
**Algorithm 1.** Fuzzification algorithm for signals from modules 1 and 2.**If**   Mean of 5 data from Module_k_ > $\frac{X_{O}+3X_{P}}{2}$     **Then**    HighN = 0, LowN = 0, Off = 0, Low = 0, Med = 0, High = 1 **Else if** $X_{P}$ < Mean of 5 data from Module_k_ ≤ $\frac{X_{O}+3X_{P}}{2}$  **Then**    Calculate the weight of Med and High by using f_2_ and f_1_ in Figure 5b    High_N_ = 0, Low_N_ = 0, Off = 0, Low = 0 **Else if** $\frac{X_{O}+X_{P}}{2}$ < Mean of 5 data from Module_k_ ≤ $X_{P}$   **Then**    Calculate the weight of Low and Med by using f_4_ and f_3_ in Figure 5b    High_N_ = 0, Low_N_ = 0, Off = 0, High = 0 **Else if** $X_{O}$ < Mean of 5 data from Module_k_ ≤ $\frac{X_{O}+X_{P}}{2}$   **Then**
    Calculate the weight of Off and Low by using f_6_ and f_5_ in Figure 5b    High_N_ = 0, Low_N_ = 0, Med = 0, High = 0 **Else if** $X_{N}\;$< Mean of 5 data from Module_k_ ≤ $X_{O}$     **Then**    Calculate the weight of Low_N_ and Off by using f8 and f7 in Figure 5b    HighN = 0, Low = 0, Med = 0, High = 0 **Else if** $\frac{3X_{N}-X_{O}}{2}\;$< Mean of 5 data from Module_k_ ≤ $X_{N}\;$   **Then**    Calculate the weight of High_N_ and Low_N_ by using f_10_ and f_9_ in Figure 5b     Off = 0, Low = 0, Med = 0, High = 0 **Else**  High_N_ = 1, Low_N_ = 0, Off = 0, Low = 0, Med = 0, High = 0 **end**(Module number: k = 1, 2)

### 3.2. Rule Evaluation

In the rule evaluation step, the names of the fuzzy values were used as inputs to the fuzzy rules to classify the forearm movements. The rules consist of conditional statements, such as ‘*if* {the fuzzy name (weight value) of the signal from module 1 and the fuzzy name (weight value) of the signal from module 2} *then* {(the strength of wrist extension motion is …), (the strength of ulnar deviation motion is …), (the strength of finger flexion motion is …), and (the strength of wrist flexion motion is …)}’. In this study, fuzzy rules were designed, where each rule determines the strength of each motion corresponding to the combinations of fuzzy names generated in the fuzzification process. Because the two signals from modules 1 and 2 generated a six-name the fuzzy state in every step, a total of 36 combinations of fuzzy rules were developed. Note that the strength of each motion in the fuzzy rules is determined only by the fuzzy names. The weight values corresponding to each fuzzy name are used in the defuzzification process. Table 1 shows an example of fuzzy rules.

### 3.3. Defuzzification

In the defuzzification step, the final defuzzification value for each motion is calculated. Because there are fuzzy names with zero weights, the only rules for which none of the fuzzy weight values correspond to the two fuzzy names for each module_1 and 2_ are zero are activated and used in the calculation of the final strength of each motion. The activated rules are categorized into five data sets, each consisting of three data columns: two columns for the weights of fuzzy names and one for the strengths of each motion in Table 1. The final strength of each motion is calculated using Equation (1) in each data set.

(1) $$x=\frac{\sum_{i=1}^{number\;of\;activated\;rules}\left(m^{i}\times w^{i}\right)}{\sum_{i=1}^{number\;of\;activated\;rules}m^{i}}\;$$

In Equation (1), ***x*** is the defuzzification value of a motion, and ***m*** is the output of the activated rules, that is, the minimum non-zero weight of the fuzzy names in the activated rules. ***w*** is the center value; two, one, and 0.5 correspond to the strengths of motions ‘High_motion_’, ‘Med_motion_’, and ‘Low_motion_’, respectively, as shown in the Table 1.

Algorithm 2 shows the MATLAB code algorithm to realize rule evaluation and defuzzification using Equation (1). The algorithm calculated all the defuzzification values of the four motions, and the motion with the maximum value was selected as the final motion of the forearm.
**Algorithm 2.** Rule evaluation and defuzzification algorithm to detect the four motions: wrist extension, ulnar deviation, finger flexion, and wrist flexion.**1. Acquiring fuzzy values consisting of names and weights from the modules 1 and 2**   Module 1: High_N1_(weight), Low_N1_(weight), Off_1_(weight), Low_1_(weight), Med_1_(weight), High_1_(weight)   Module 2: High_N2_(weight), Low_N2_(weight), Off_2_(weight), Low_2_(weight), Med_2_(weight), High_2_(weight) **2. Defuzzification process using (1)**
 **(1) Calculation of numerator in (1):**  WE_num_ = MIN (High_N1_, High_N2_)Rule1 × High_motion_(=2) + ···        + MIN (Low_1_, Off_2_)Rule21 × Low_motion_(=0.5) + ··· + MIN (High_1_, High_2_)Rule36 × Low_motion_(=0.5)  UD_num_ = MIN (High_N1_, High_N2_)Rule1 × Low_motion_(=0.5) + ···        + MIN (Low_1_, Off_2_)Rule21 × High_motion_(=1) + ··· + MIN (High_1_, High_2_)Rule36 × Low_motion_(=0.5)  FF_num_ = MIN (High_N1_, High_N2_)Rule1 × Low_motion_(=0.5) + ···        + MIN (Low_1_, Off_2_)Rule21 × Low_motion_(=1) + ··· + MIN (High_1_, High_2_)Rule36 × Low_motion_(=0.5)  WF_num_ = MIN (High_N1_, High_N2_)Rule1 × Low_motion_(=0.5) + ···        + MIN (Low_1_, Off_2_)Rule21 × Low_motion_(=0.5) + ··· + MIN (High_1_, High_2_)Rule36 × High_motion_(=0.5)  OS_num_ = MIN (High_N1_, High_N2_)Rule1 × Low_motion_(=0.5) + ···        + MIN (Low_1_, Off_2_)Rule21 × Med_motion_(=0.5) + ··· + MIN (High_1_, High_2_)Rule36 × Low_motion_(=2) **(2) Calculation of denominator in (1):**  WE_den_ = MIN (High_N1_, High_N2_)Rule1 + ··· + MIN (Low_1_, Off_2_)Rule21 + ··· + MIN (High_1_, High_2_)Rule36  UD_den_ = MIN (High_N1_, High_N2_)Rule1 + ··· + MIN (Low_1_, Off_2_)Rule21 + ··· + MIN (High_1_, High_2_)Rule36  FF_den_ = MIN (High_N1_, High_N2_)Rule1 + ··· + MIN (Low_1_, Off_2_)Rule21 + ··· + MIN (High_1_, High_2_)Rule36  WF_den_ = MIN (High_N1_, High_N2_)Rule1 + ··· + MIN (Low_1_, Off_2_)Rule21 + ··· + MIN (High_1_, High_2_)Rule36  OS_den_ = MIN (High_N1_, High_N2_)Rule1 + ··· + MIN (Low_1_, Off_2_)Rule21 + ··· + MIN (High_1_, High_2_)Rule36  **(3) Calculation of defuzzification vlaues for each motion:**  Defuzzification value for wrist extension = WE_num_/WE_den_ = α  Defuzzification value for wrist extension = UD_num_/UD_den_ = β  Defuzzification value for ulnar deviation = FF_num_/FF_den_ = γ  Defuzzification value for finger flexion = WF_num_/WF_den_ = δ  Defuzzification value for wrist flexion = OS_num_/OS_den_ = ε **(4) Decision of the motion of forearm:**
  The motion of forearm = MAX[α β γ δ ε]

## 4. Results and Discussion

**Figure 6:**
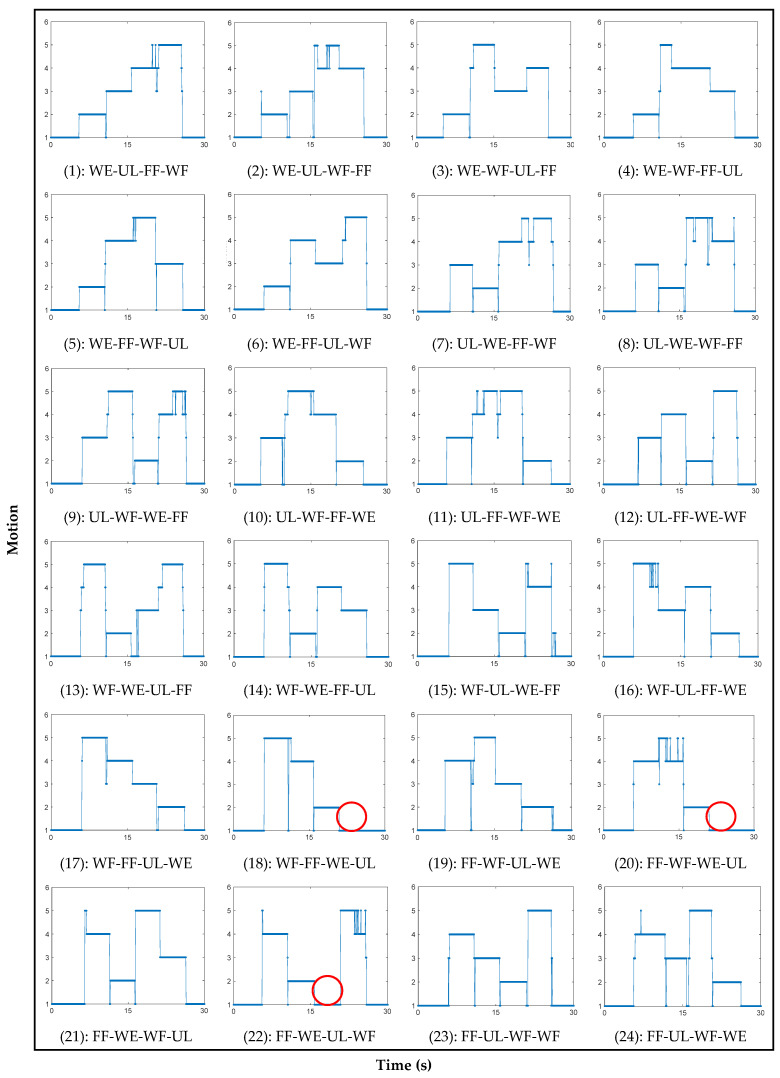
Detection results of 24 consecutive motions of forearm (WE: wrist extension, UL: ulnar deviation, FF: finger flexion, and WF: wrist extension).

Figure 6 shows the detected result of 24 sets of motions consisting of all the combinations of the four motions using the measured signals and fuzzy algorithm.

The intention of the motions is expressed using the points on the graph as follows: ‘1’ for off state, ‘2’ for wrist extension, ‘3’ for ulnar deviation, ‘4’ for finger flexion, and ‘5’ for wrist flexion.

The results show that the fuzzy algorithm effectively detects all consecutive motions of the forearm. However, some failures occurred while detecting these motions. All detection failures occurred when the motion was changed from wrist extension to ulnar deviation, as shown in Figure 6 (18), (20), and (22). The red circles in Figure 6 indicate the locations where detection failures occurred. Table 2 shows the accuracy rate of each motion where Accuracy rate_1_ and Accuracy rate_2_ represent the success percentiles of motion detection with and without including the three detection failure cases, respectively.

These results show that, when the developed fuzzy logic algorithm was applied on the motion detection of ‘wrist extension’ and ‘ulnar deviation’ motions, approximately 100% detection accuracies were obtained as shown in the second row in Table 2. In addition, the detection accuracies of the ‘off state’ were approximately 100%. Moreover, the detection accuracies of ‘finger flexion’ and ‘wrist flexion’ motions were close to 90%, which was similar or higher than the accuracy rates of other studies that applied neural networks and fuzzy logic using sEMG [3,4,36,37,39,42]. To adjust the connection strength within the internal network, the neural network theory, which is a learning approach based on repetition, must be conducted in advance. In general, as the number of learning iterations increases, the error between the command signal and the actual movement decreases; here, the final mean accuracy rates were close to 95% [3,4,36,37].

The advantages of the developed sensor are as follows. First, the patch sensors used in the sEMG method, which would cover most parts of the forearm, were substituted with a single thin bracelet-type sensor that could be worn around the forearm. As a result, compared to the conventional sEMG technique, the number of sensors in contact with the skin surface was reduced by more than 70%. Second, the developed bracelet sensor has the thinnest size compared to other band type sensors that were reported [3,6,11,12,22,23,24,43] because the width of the module, which is the largest width of the sensor, is only 12 mm, with a thickness of 5 mm. Therefore, the proposed device should be comfortable to wear and easy to use in practical applications. Third, the key materials of the sensor are PET film and conductive carbon paste, which enable low-cost fabrication compared to other sensors using CNTs [30,31,32,33,34]. Although the thin PET film is fragile, it was safely bent under the guidance of the mechanical parts of the sensor module by avoiding excessive compressive forces due to undesirable bending motions, such as opposite directional bending, folding, and twisting. Moreover, the convenience of handling of the PET film and carbon paste makes it possible to fabricate smaller sensors. Finally, because the simple bending motion of the polymer film produces a signal with high sensitivity, the proposed method has promising applications in wearable sensor technology for detecting body movements. They can also be widely applied to various types of mechanical guides.

Although the developed bracelet sensor exhibited promising performance, there are some drawbacks that must be improved before it can be practically applied. In this study, two sensor modules were placed on the surface area of the forearm muscle groups: extensor carpi ulnaris and flexor carpi ulnaris. If the location of the sensor module varies, the pattern of the signals will vary, resulting in a decrease in the efficiency of motion detection. Therefore, future research will focus on the investigation of techniques to produce constant performance without considering the location of the sensor modules of the bracelet sensor, such as the recognition of the shape variation of the entire forearm circumference, where the sensor is placed by using multiple sensor modules. In addition, the transducer circuit will be integrated into the bracelet sensor with a more downsized sensor module design.

## 5. Conclusions

This study developed a fuzzy logic algorithm to detect the four motions of the forearm using the signals from the bracelet sensor, which was developed in a previous study [25]. The fuzzy algorithm successfully detected four motions and the resting state of the forearms. Compared with the results of previous research, two issues were improved in this study. First, herein, two sensor modules were used, which showed successful results in detecting motions of the forearm, whereas, in the previous research, the bracelet sensor with three sensor modules detected the motion more effectively. Second, in this research, 24 cases of four consecutive motions of the forearm were detected using the designed fuzzy logic algorithm, whereas, in the previous research, the motion detection experiment was conducted only for each separate motion. Although the overall accuracy rate was 94%, the accuracy rates for the resting state, wrist extension, and ulnar deviation were approximately 100%. However, detection failure was observed in three cases, as shown in Figure 6 (18), (20), and (22), because the relative variation in the surface area in contact with the sensor module is very small, whereas the forearm changed its motion from wrist extension to ulnar deviation.

The developed bracelet sensor and motion detection algorithm provided more comfortable wearing and ease of practical use. Future work will be conducted to overcome the drawbacks observed during the study and to apply it for practical use. 

## Figures and Tables

**Table 1:** Summary of fuzzy rules.

Rules	If	Fuzzy Names			Then	Strength of Motions				
		Module_1_	and	Module_2_		Wrist Extension	Ulnar Deviation	Finger Flexion	Wrist Flexion	Off State
1	***If***	High_N_	***and***	High_N_	***Then***	High_motion_	Low_motion_	Low_motion_	Low_motion_	Low_motion_
2		High_N_		Low_N_		Med_motion_	Low_motion_	Low_motion_	Low_motion_	Low_motion_
3		High_N_		Off		Low_motion_	Low_motion_	Low_motion_	Low_motion_	Low_motion_
⁝		⁝		⁝		⁝				
7		Low_N_		High_N_		Med_motion_	Low_motion_	Low_motion_	Low_motion_	Low_motion_
8		Low_N_		Low_N_		Med_motion_	Low_motion_	Low_motion_	Low_motion_	Med_motion_
9		Low_N_		Off		Low_motion_	Low_motion_	Low_motion_	Low_motion_	Med_motion_
⁝		⁝		⁝		⁝				
13		Off		High_N_		Low_motion_	Low_motion_	Low_motion_	Low_motion_	Low_motion_
14		Off		Low_N_		Low_motion_	Low_motion_	Low_motion_	Low_motion_	Med_motion_
15		Off		Off		Low_motion_	Low_motion_	Low_motion_	Low_motion_	High_motion_
⁝		⁝		⁝		⁝				
21		Low		Off		Low_motion_	High_motion_	Low_motion_	Low_motion_	Med_motion_
22		Low		Low		Low_motion_	Med_motion_	Low_motion_	Low_motion_	Med_motion_
23		Low		Med		Low_motion_	Low_motion_	Low_motion_	Low_motion_	Low_motion_
⁝		⁝		⁝		⁝				
28		Med		Low		Low_motion_	Med_motion_	Low_motion_	Low_motion_	Low_motion_
29		Med		Med		Low_motion_	Low_motion_	Med_motion_	Low_motion_	Low_motion_
30		Med		High		Low_motion_	Low_motion_	High_motion_	Low_motion_	Low_motion_
⁝		⁝		⁝		⁝				
34		High		Low		Low_motion_	Low_motion_	Low_motion_	Low_motion_	Low_motion_
35		High		Med		Low_motion_	Low_motion_	Med_motion_	Med_motion_	Low_motion_
36		High		High		Low_motion_	Low_motion_	Low_motion_	High_motion_	Low_motion_

**Table 2:** Motion detection errors.

Motion	Resting State	Wrist Extension	Ulnar Deviation	Finger Flexion	Wrist Flexion
Accuracy rate_1_ (%)	99.80	99.79	85.67	87.88	84.43
Accuracy rate_2_ (%)	99.80	99.75	98.52	86.68	87.34
